# Discordance Between Patient and Caregiver on Ratings of Purpose in Life Following a Dementia Diagnostic Appointment

**DOI:** 10.1093/geroni/igaa057.909

**Published:** 2020-12-16

**Authors:** Matthew Wynn, Catherine Ju, Patrick Hill

**Affiliations:** 1 Washington University in St. Louis, St. Louis, Missouri, United States; 2 Washington University in St. Louis, Saint Louis, Missouri, United States

## Abstract

Purpose in life has been linked with better well-being and reduced risk for major illness. As such, work has focused on understanding what leads to changes in sense of purpose during adulthood, with a focus on major life events. Receiving a dementia diagnosis is a major life event that could affect purpose in life both for older adults with dementia and their potential caregivers. To examine this issue participants answered questions at two timepoints, before their diagnostic appointment at a specialized memory clinic, and between two days and two weeks after the appointment. Participants provided self-report ratings of sense of purpose and as well as open-ended answers regarding their purpose in life. Data is available from both caregivers and patients and qualitative coding was performed on participants’ open-ended responses. Our analysis revealed discordance between patients and caregivers, such that caregivers’ ratings of patients were lower in terms of purpose in life (t = -5.63, p < .001) and being in worse health (t = -3.41, p < .001) than patients’ ratings of themselves on the same measures. Discordance between caregiver and patient in the context of a dementia diagnostic appointment and outcomes associated with this discordance are discussed.

